# Rehabilitation strategies for long COVID: integrating human factors engineering

**DOI:** 10.3389/fresc.2026.1708460

**Published:** 2026-02-12

**Authors:** Tzu-Sui Hung

**Affiliations:** Department of Mechanical and Industrial Engineering, Vanung University, Taoyuan, Taiwan

**Keywords:** long COVID, rehabilitation, human factors engineering, adaptive strategies, patient-centered

## Abstract

Long COVID presents unique challenges that extend beyond conventional biomedical rehabilitation, necessitating strategies that are adaptive, multidisciplinary, and patient-centered. This mini-review synthesizes current evidence on physical, cognitive, and occupational rehabilitation, and introduces human factors engineering as a framework to optimize the design, delivery, and usability of interventions. Emerging approaches such as telerehabilitation, cognitive ergonomics, and structured return-to-work programs illustrate the value of integrating clinical rehabilitation with user-centered design. Yet critical gaps remain, including the limited number of randomized controlled trials, the heterogeneity of outcome measures, and the lack of systematic integration between rehabilitation and human factors research. Addressing these challenges will be essential to develop effective, scalable, and sustainable interventions. By aligning rehabilitation protocols with the principles of human factors engineering, future practice can better enhance efficacy, promote sustained patient engagement, and ultimately improve the quality of life for individuals living with long COVID.

## Introduction

Human factors engineering (HFE) provides a critical framework for reassessing and enhancing rehabilitation strategies for long COVID. HFE examines how people interact with systems, environments, and technologies, emphasizing the optimization of safety, usability, efficiency, and human well-being. In rehabilitation, these principles aim to design programs that accommodate human variability and minimize cognitive and physical burdens on patients. Beyond conventional biomedical approaches, HFE emphasizes the interaction between patients and their physical, cognitive, and social environments, promoting the development of interventions that are adaptive, intuitive, and sustainable, ensuring that patients can engage safely and meaningfully throughout the recovery process (1).

Applying HFE principles to long COVID is particularly relevant given the condition's fluctuating symptom patterns, multi-system involvement, and extended recovery trajectories. By focusing on patient–environment interaction and feedback, HFE encourages rehabilitation programs that adapt to individual pacing, energy capacity, and tolerance levels, rather than adhering to rigid performance metrics. This approach aligns with patient-centered rehabilitation philosophies and provides clinicians with structured yet flexible frameworks to prevent post-exertional malaise (1, 2).

Patient-centered and adaptive strategies are essential. Interventions should prioritize disability mitigation and functional optimization over rigid outcome targets, emphasizing energy conservation, pacing, and symptom-titrated activity to reduce the risk of post-exertional malaise (1, 2). Multidisciplinary programs that integrate physical, cognitive, and psychosocial therapies have shown promise in improving patient well-being and functional capacity (3–5). The episodic and unpredictable nature of long COVID highlights the need for flexible rehabilitation models that align with the concept of episodic disability, supporting self-management and day-to-day adaptation (6).

Traditional exercise-based interventions remain contentious due to the risk of post-exertional symptom exacerbation, particularly in individuals with overlapping conditions such as myalgic encephalomyelitis/chronic fatigue syndrome or postural orthostatic tachycardia syndrome (7, 8). Emerging evidence supports structured pacing protocols and micro-choice–based approaches, which allow patients to make incremental, autonomous decisions about activity levels while maintaining control and minimizing symptom flares (9, 10).

A comprehensive approach must also include careful screening for autonomic dysfunction, orthostatic intolerance, or cardiac impairment before initiating activity-based interventions (11, 12). Clinicians should avoid terminology or recommendations that may inadvertently encourage unsafe exertion and instead adopt communication strategies that reinforce patient safety. Ultimately, effective rehabilitation for long COVID requires an in-depth understanding of its physiological, cognitive, and psychological impact, emphasizing energy management, pacing, and symptom-contingent activity as central pillars of care (13). Teaching patients to recognize and remain within their individual “energy envelope” has proven particularly valuable in preventing relapse and supporting sustainable recovery (14).

In summary, integrating HFE principles into long COVID rehabilitation supports the creation of safer, more usable, and adaptive interventions that align with patient variability and recovery capacity. The following sections address physical, cognitive, telerehabilitation, and vocational strategies, followed by discussion and directions for future research

## Physical rehabilitation

The multifactorial nature of long COVID symptoms requires individualized and adaptive rehabilitation programs that reflect fluctuating severity and diverse manifestations (8). This approach moves beyond one-size-fits-all protocols by emphasizing nuanced assessment of symptom burden and its impact on daily functioning (15). Traditional models relying on standardized, progressively challenging exercise can be detrimental for patients with post-exertional malaise (7). Instead, rehabilitation should incorporate real-time symptom feedback and prioritize energy conservation to minimize exacerbations (16).

Persistent fatigue, often overlapping with myalgic encephalomyelitis/chronic fatigue syndrome, necessitates cautious implementation of pacing strategies to avoid symptom flares (8). Structured pacing protocols, supported by activity diaries and self-monitoring tools, empower patients to regulate exertion, identify triggers, and sustain gradual recovery (10, 16). Therefore, this pacing-centered approach—aimed at preventing post-exertional malaise—is increasingly recognized as foundational in long COVID rehabilitation (41). In fact, multidisciplinary interventions that combine exercise modification with sleep, nutrition, and psychological support offer additional benefits for fatigue and dyspnea management (17).

In summary, physical rehabilitation for long COVID should adopt adaptive, pacing-based frameworks that respect symptom variability, prioritize patient safety, and integrate holistic support to enhance recovery.

## Cognitive rehabilitation

Cognitive rehabilitation targets impairments such as brain fog, attention deficits, and memory loss, using both restorative exercises and compensatory strategies. Cognitive ergonomics principles further optimize task design and environmental conditions, reducing cognitive load and improving performance (2). Techniques include metacognitive strategy training, attention processing training, and environmental adaptations such as checklists, workspace organization, and assistive technologies (18).

Evidence supports the benefits of cognitive stimulation and telerehabilitation platforms, which improve accessibility, flexibility, and continuity of care, particularly for patients with mobility or geographic barriers (19, 42). Integrated approaches combining retraining with environmental adjustments enhance independence and daily functioning (15).

Neurocognitive symptoms—including executive dysfunction, impaired memory, and reduced processing speed—mirror sequelae seen in other post-viral syndromes and are prevalent in more than 20% of patients even months after infection (43). Early identification and tailored interventions, including Constraint-Induced Cognitive Therapy, have demonstrated promising results in improving daily functioning (20, 21). Ongoing monitoring with both objective measures and patient-reported outcomes is essential. Mechanistic studies point to viral neuroinvasion, neuroinflammation, microvascular injury, and hypoxia as potential contributors, highlighting the need for biomarker development to support precision rehabilitation strategies (17).

In summary, cognitive rehabilitation should integrate ergonomic design, metacognitive training, and environmental adaptation to reduce cognitive burden and support functional independence in patients recovering from long COVID.

## Telerehabilitation: design challenges and human–computer interaction principles

Telerehabilitation, as part of information and communication technologies (ICT), has rapidly expanded during the pandemic, has shown its potential but also exposed challenges related to equity, usability, and data security. Human-centered design and human–computer interaction principles are essential to ensure that digital rehabilitation tools remain intuitive, accessible, and inclusive, particularly for individuals with cognitive or physical limitations (22).

Artificial intelligence (AI) can further personalize care, monitor patient progress, and optimize outcomes, though risks such as algorithmic bias and digital disparities must be carefully managed (23, 24). “Rehabilomics” approaches that integrate multi-omic data with clinical phenotypes may enable precision rehabilitation (25). Similar frameworks have been successfully applied in neurodegenerative conditions and could be adapted for long COVID (26).

Future telerehabilitation platforms must address variable symptom presentation through adaptive, individualized programming, potentially augmented by machine learning and mobile apps (27). Such technology-driven programs can enhance flexibility, promote sustained engagement, and support multidisciplinary care delivery.

In summary, telerehabilitation represents an important ICT-based strategy for long COVID management, combining accessibility, personalization, and real-time monitoring under human-centered and ethical design principles.

## Vocational rehabilitation and return-to-work

Supporting a safe and sustainable return to work is a central, yet complex, aspect of long COVID rehabilitation. This requires vocational assessments, ergonomic workplace modifications, and phased return-to-work programs tailored to fluctuating symptoms (28). Integrating cognitive-behavioral interventions and energy management can address barriers such as fatigue and brain fog (29).

Collaboration among healthcare providers, employers, and vocational specialists is vital for adaptive planning, while digital tools such as wearables and AI-driven monitoring may help track symptoms and guide adjustments (18). Occupational therapists play a key role in evaluating job-related functional capacity and designing accommodations (30).

Conventional return-to-work models assume linear recovery, which often conflicts with the episodic and unpredictable course of long COVID (31). Alternative frameworks such as Universal Design and the International Classification of Functioning, Disability and Health can guide inclusive workplace practices and rehabilitation planning (32). Even after inpatient rehabilitation, reduced work ability remains common (33, 34), underscoring the need for flexible, interdisciplinary approaches that extend beyond traditional timelines and view return to work as an ongoing process rather than a discrete endpoint.

Therefore, vocational rehabilitation for long COVID should emphasize flexible, inclusive, and ongoing workplace reintegration supported by interdisciplinary collaboration and patient-centered planning.

## Discussion

The protracted and multifaceted challenges associated with long COVID necessitate a rehabilitation framework that extends beyond conventional models. Integrating human factors engineering (HFE) principles offers a pathway to optimize intervention design, delivery, and usability, while shifting the focus toward patient-centered outcomes and real-world functionality. Given the heterogeneity of symptoms and recovery trajectories, individualized rehabilitation pathways are essential for effective management (30, 35, 39).

Rehabilitation must be reframed as a process that mitigates disability and optimizes function rather than aiming solely for cure. Psychological support is equally important to counteract stigma and disbelief frequently encountered by patients (2). This calls for interdisciplinary models that integrate physical, cognitive, and psychosocial interventions, tailored to the constellation of symptoms presented by each individual. Strategies for self-management and adaptive coping mechanisms further empower patients to navigate fluctuating conditions (3, 36).

Advanced digital tools such as telerehabilitation and AI-assisted monitoring can extend care beyond traditional clinical settings, providing adaptive and scalable interventions (37). However, these innovations must ensure usability, data security, and accessibility to maintain equity and trust. Rehabilitation must also address socioeconomic and environmental factors, including workplace reintegration and social participation (30). Fatigue management remains a cornerstone of all pathways, requiring integrated strategies to support work ability and daily functioning (38).

In summary, an HFE-informed rehabilitation model should prioritize usability, interdisciplinary collaboration, and adaptability to enhance the sustainability and effectiveness of long COVID recovery programs

## Future research

Long COVID rehabilitation remains an emerging field, and several priorities for future research and practice are clear. First, well-designed randomized controlled trials are urgently needed to evaluate interventions tailored to fluctuating, post-viral conditions. Current approaches often draw on evidence from other syndromes, such as chronic fatigue syndrome or post-intensive care rehabilitation, without sufficient validation in long COVID populations.

Second, HFE principles should be systematically integrated into clinical trial design and program development. Embedding usability, workload, and accessibility assessments will help ensure that interventions are not only effective but also feasible and scalable in diverse real-world contexts. Patient co-design approaches represent an important next step to improve ecological validity and long-term engagement.

Third, the role of digital health technologies—including telerehabilitation and AI-enhanced platforms—requires rigorous evaluation. While these tools offer scalability and personalization, future studies must address their clinical effectiveness, equity of access, and data security. Digital disparities, particularly related to internet access and digital literacy, must be considered to avoid exacerbating health inequities.

Fourth, longitudinal studies are needed to map recovery trajectories and identify predictors of long-term disability vs. functional recovery. Biomarker research could further clarify biological underpinnings and inform precision rehabilitation.

Finally, policy-oriented research is required to address structural barriers, including workforce shortages, fragmented care delivery, and limitations in insurance coverage. Developing integrated care pathways, multidisciplinary “one-stop” clinics, and adaptive return-to-work models will be crucial for reducing disability burden and socioeconomic impact.
